# Neurotoxic Sleight of Fang: Differential Antivenom Efficacy Against Mamba (*Dendroaspis* spp.) Venom Spastic-Paralysis Presynaptic/Synaptic vs. Flaccid-Paralysis Postsynaptic Effects

**DOI:** 10.3390/toxins17100481

**Published:** 2025-09-26

**Authors:** Lee Jones, Mimi Lay, Lorenzo Seneci, Wayne C. Hodgson, Ivan Koludarov, Tobias Senoner, Raul Soria, Bryan G. Fry

**Affiliations:** 1 Adaptive Biotoxicology Lab, School of the Environment, The University of Queensland, St. Lucia, QLD 4072, Australia; lee.jones1@student.uq.edu.au (L.J.); l.seneci@student.uq.edu.au (L.S.); 2 Monash Venom Group, Department of Pharmacology, Biomedical Discovery Institute, Monash University, Clayton, VIC 3800, Australia; mimi.lay@monash.edu (M.L.); wayne.hodgson@monash.edu (W.C.H.); 3 Institute for Insect Biotechnology, Heinrich-Buff-Ring 58, 35392 Giessen, Germany; jcoludar@gmail.com; 4 Department of Informatics, Boinformatics, and Computational Biology, i12, Technical University of Munich, Boltzmannstraße 3, Garching, 85748 Munich, Germany; tobias.senoner@tum.de; 5 Inosan Biopharma, 28001 Madrid, Spain; rsoria@inosanbiopharma.com

**Keywords:** *Dendoaspis*, neurotoxic, evolution, antivenom, paralysis

## Abstract

Mamba (*Dendroaspis* species) snakebites are critical medical emergencies across sub-Saharan Africa. Envenomings can result in the rapid onset of complex neurotoxic symptoms, often leading to high rates of mortality without timely intervention with antivenom. The ancestral state of mambas is the green coloured, forest dwelling type, with the tan/grey coloured, savannah dwelling *D. polylepis* (Black Mamba) representing a derived state both ecologically and morphologically. However, it has not been tested whether these changes are paralleled by changes in venom biochemistry or if there are differential molecular evolutionary patterns. To fill these knowledge gaps, this study evaluated the neurotoxic effects of all *Dendroaspis* species venoms using the chick biventer cervicis nerve-muscle preparation, assessed the neutralizing efficacy of three antivenoms commercially available in Africa, and reconstructed the molecular evolutionary history of the toxin types to ascertain whether some were unique to particular species. All *Dendroaspis* venoms demonstrated potent flaccid-paralysis due to postsynaptic neurotoxicity. The only exception was *D. angusticeps* venom, which conversely exhibited spastic-paralysis due to presynaptic/synaptic neurotoxicity characterised by potentiation of acetylcholine presynaptic release and sustained synaptic activity of this neurotransmitter. Antivenom efficacy varied significantly. All three antivenoms neutralized to some degree the flaccid-paralysis postsynaptic effects for all species, with *D. viridis* venom being the best neutralized, and this pattern extended to all the antivenoms. However, neutralisation of flaccid-paralysis postsynaptic effects unmasked spastic-paralysis presynaptic/synaptic neurotoxicity within non-*angusticeps* venoms. Spastic-paralysis presynaptic effects were poorly neutralized for all species by all antivenoms, consistent with prior clinical reports of poor neutralisation of spastic-paralytic effects. Geographic variation in *D. polylepis* venom was evident for the relative neutralisation of both spastic-paralysis presynaptic/synaptic and flaccid-paralysis postsynaptic/synaptic neurotoxic pathophysiological effects, with differential neutralization capabilities noted between the Kenyan and South African populations studied. Molecular phylogenetic analyses confirmed spastic-paralysis and flaccid- paralysis toxins to be a trait that emerged in the *Dendroaspis* last common ancestor, with all species sharing all toxin types. Therefore, differences in venoms’ pathophysiological actions between species are due to differential expression of toxin isoforms rather than the evolution of species-specific novel toxins. Our findings highlight the synergistic nature of flaccid-paralysis postsynaptic and spastic-paralysis presynaptic/synaptic toxins, while contributing significant clinical and evolutionary knowledge of *Dendroaspis* venoms. These data are crucial for the continued development of more effective therapeutic interventions to improve clinical outcomes and for evidence-based design of clinical management strategies for the envenomed patient.

## 1. Introduction

Snakebite envenoming represents a significant public health crisis across sub-Saharan Africa, where approximately 500,000 envenomings occur resulting in 30,000 deaths per year [1,2]. However, this is likely underestimated, due to the lack of reliable epidemiological and health data. Therefore, there is a need to establish the effectiveness of available treatments against the functional aspects of snake venoms.

Snakes from the *Dendroaspis* genus are widely distributed across sub-Saharan Africa, and there are currently four recognised species: the *Dendroaspis angusticeps* (Eastern Green Mamba); *Dendroaspis jamesoni* (Jameson’s Mamba), consisting of the two subspecies *D. j. jamesoni* (Western Jameson’s Mamba) and the *D. j. kaimosae* (Eastern Jameson’s Mamba); *Dendroaspis Polylepis* (Black Mamba); and *Dendroaspis viridis* (Western Green Mamba) [3]. Notably, the green mambas do not form a natural group; instead, the phylogenetic patterning for the genus is (*D. angusticeps* + *D. polylepis*) + (*D. jamesoni* + *D. viridis*). As the *D. polylepis* renders the green mamba species non-monophyletic, this suggests that the ancestral phenotype was a green coloured forest dweller, with the savannah dwelling *D. polylepis* representing a derived ecological and morphological state.

Each of these four species is medically significant due to the rapid onset and severity of envenoming. *D. polylepis* in particular is recognised by the World Health Organization as a significant medical concern due to its highly potent venom, evolution as a swift terrestrial predator, and as it is an adaptable species for disturbed habitats it has an extensive distribution across populated and agricultural areas, which increases the likelihood of potentially fatal interactions [4]. All *Dendroaspis* species possess a potent neurotoxic venom that can rapidly induce fasciculations, cardiovascular collapse, and respiratory paralysis in humans, with death potentially occurring within 45 min in extreme cases [5,6]. Without prompt administration of mechanical ventilation and antivenom, fatality rates remain critically high [5,7].

Diverse neurotoxins are well described in *Dendroaspis* species, with proteomic studies revealing a distinctive composition characterised by the prevalence of three finger toxins (3FTx) and *Dendroaspis*-specific kunitz-scaffold peptides, known as dendrotoxins [8,9,10,11]. The neurotoxic effects of *Dendroaspis* venoms result from any one of or combination of three principal toxin classes, each targeting a distinct component of neuromuscular signalling. Spastic-paralysis is the result of either excessive release of a neurotransmitter, or the prevention of neurotransmitter degradation. Conversely, flaccid-paralysis is the result of either prevention of neurotransmitter release or the blockade of neurotransmitter receptors on the muscle. Spastic-paralysis results from kunitz-toxins (dendrotoxins) that block voltage-dependent potassium channels, leading to a prolongation of action potential duration and increased acetylcholine release into the synapse [12]. Muscle spasms are also produced by 3FTx toxins (fasciculins), which inhibit the regulatory enzyme acetylcholinesterase, leading to sustained elevations in levels of the neurotransmitter acetylcholine [13]. Flaccid-paralysis is the result of α-neurotoxins that act postsynaptically by competitively binding to nicotinic acetylcholine receptors, thereby blocking acetylcholine from initiating muscle contraction [14]. Cardiac contractility is depressed through 3FTx (calciseptins) and kunitz-toxins (calcicludines), which have convergently evolved to bind to calcium channels and block the L-type current of cardiac cells [15,16]. Myriad other neurotoxin types have been documented, including 3FTx, which block adrenergic (adrenoceptors) and cholinergic (muscarinic) receptors [17,18,19,20,21,22,23,24]. The synergistic activity of these diverse neurotoxins within *Dendroaspis* venoms drives potency and clinical severity, resulting in an unusually rapid onset of potentially lethal symptoms [10]. However, despite the novelty and diversity of neurotoxins in *Dendroaspis* toxins, their molecular evolution has not been comprehensively examined to date.

Given the medical importance of *Dendroaspis* snakes, their venoms are often included in the immunising mixtures used to produce polyspecific antivenoms for sub-Saharan Africa. Currently, eight polyvalent antivenoms are commercially available for this region [8,25]. However, neutralisation studies have demonstrated variable efficacy across different *Dendroaspis* species [8,26]. Notably, the venom of *D. angusticeps* poses a particular challenge due to its unique composition. Unlike other *Dendroaspis* species, which have venoms dominated by 3FTx, *D. angusticeps* venom is rich in kunitz-toxins [8,10]. This distinct venom composition may result in differential cross-neutralisation by antivenoms raised primarily against the venoms of α-neurotoxin-rich species [8]. The variation in cross-reactivity patterns observed across different *Dendroaspis* species underscores the need to further test antivenoms against all members of the genus. As such, the aim of this study was to characterise the neurotoxic effects of various *Dendroaspis* species’ venoms from different localities and assess the ability of three selected commercial antivenoms to neutralise the toxicity of whole venom, and to relate these variations to the molecular evolutionary patterns of the toxins.

## 2. Results

### 2.1. Neurotoxicity and Antivenom Testing

**Figure 1:**
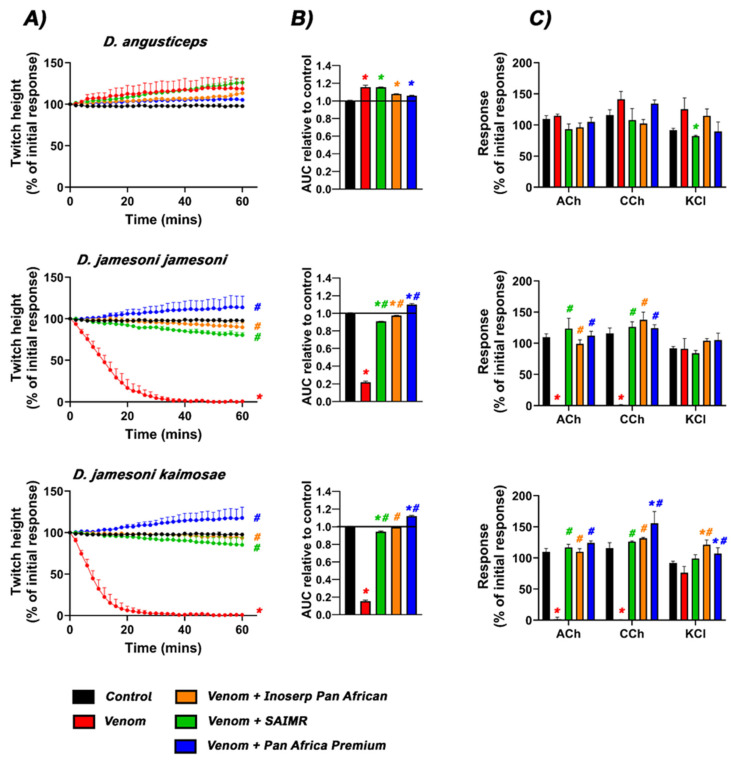
Effects of venoms (30 µg/mL) from *D. angusticeps*, *D. jamesoni jamesoni*, and *D. jamesoni kaimosae* in the presence and absence of Inoserp Pan African (orange), SAIMR (green), and Pan Africa premium (blue) antivenom on (**A**) indirect twitch height, (**B**) Area under the curve (AUC) of twitch height, and (**C**) contractile responses to agonist administrations. * Indicates significant difference to the control (*p*  <  0.05); # indicates venom alone is significantly different to venom in the presence of antivenom (*p*  <  0.05). All venom experiments were performed in quadruplicate (*n*  =  4), and venom  +  antivenom experiments in triplicate (*n*  =  3).

**Figure 2:**
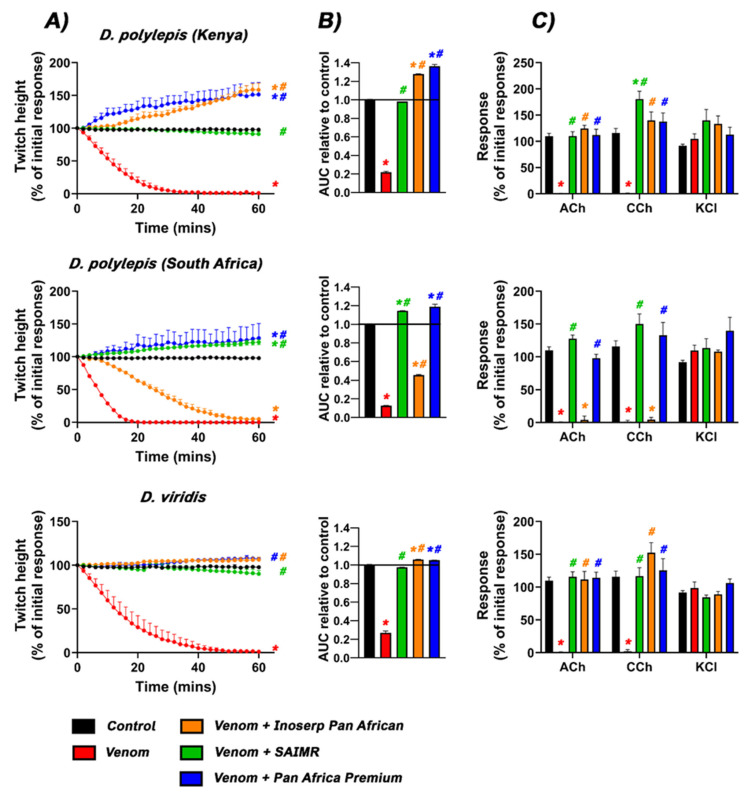
Effects of venoms (30 µg/mL) from *D. polylepis* (Kenya), *D. polylepis* (South Africa), and *D. viridis* in the presence and absence of Inoserp Pan African (orange), SAIMR (green), and Pan Africa Premium (blue) antivenom on (**A**) indirect twitch height, (**B**) Area under the curve (AUC) of twitch height, and (**C**) contractile responses. * Indicates significant difference to the control (*p*  <  0.05); # indicates venom alone is significantly different to venom in the presence of antivenom (*p*  <  0.05). All venom experiments were performed in quadruplicate (*n*  =  4), and venom  +  antivenom experiments in triplicate (*n*  =  3).

Venoms from all species, except *D. angusticeps*, displayed potent flaccid-paralysis postsynaptic neurotoxicity as indicated by the abolishment of twitches in 30 min or less (Figure 1A and Figure 2A). The site of action was confirmed by the abolishment of the acetylcholine (Ach) and carbamylcholine (CCh) responses, with no significant effect on the contractile response to potassium chloride (KCl) (Figure 1C and Figure 2C). The ACh/CCh response is due to the nicotinic acetylcholine receptors (NAChR) being blocked while, conversely, the KCl response bypasses NAChR to act directly on the muscle. This confirms that the twitch abolishment is due to NAChR-blockage, not due to muscle fibre damage. The relative potency of flaccid-paralysis postsynaptic neurotoxicity is shown in Table 1. In contrast, *D. angusticeps* displayed a spastic-paralysis effect, increasing twitch height over the course of the assay (Figure 1A).

Reflective of the variances in immunizing mixtures, the three antivenoms displayed differential efficacy in preventing neurotoxic outcomes (Figure 1A,B and Figure 2A,B). Each antivenom offered only partial protection for some venoms (good for SAIMR against *D. polylepis* (Kenya), *D. viridis*, *D. j. jamesoni* and *D. j. kaimosae*), while proving inefficacious against others. The spastic-paralysis presynaptic/synaptic effect of *D. angusticeps* venom was neutralised (in rank order) by Pan African Premium > Inoserp > SAIMR polyvalent. Conspicuously, antivenoms unmasked background spastic-paralysis presynaptic/synaptic neurotoxicity in non-*angusticeps* venoms. Our results also showed that no antivenom displayed full protection of all venom activities against the South African locality of *D. polylepis*.

The Pan African Premium antivenom protected against the flaccid-paralysis postsynaptic neurotoxic effects from all *Dendroaspis* venoms and prevented the abolishment of contractile responses to ACh and CCh. However, the antivenom was unable to neutralise the spastic-paralysis presynaptic/synaptic effects (characterised by an increase in twitch height after venom addition) caused by *D. angusticeps*, both localities of *D. polylepis* (Kenya and South Africa), *D. j. jamesoni* and *D*. *j. kaimosae* venoms.

The Inoserp Pan African antivenom partially delayed the flaccid-paralysis postsynaptic-neurotoxic effects of venom from *D. polylepis* (South Africa) but did not restore responses to exogenous agonists ACh or CCh, indicating residual flaccid-paralysis postsynaptic binding of toxins. In contrast, the Inoserp antivenom protected against the postsynaptic effects of the Kenyan *D. polylepis* sample but not against the spastic-paralysis presynaptic/synaptic effects. As such, there was an increase in neuromuscular activity, i.e., twitch height. In contrast, both spastic-paralysis presynaptic/synaptic and flaccid-paralysis postsynaptic neurotoxic effects from *D. j. jamesoni*, and *D. j. kaimosae* were successfully neutralised by the Inoserp Pan African antivenom, maintaining twitches that were not significantly different to the control.

SAIMR polyvalent antivenom successfully protected against the flaccid-paralysis postsynaptic effects of *D. viridis*, as well as both the spastic-paralysis presynaptic/synaptic and flaccid-paralysis postsynaptic effects from *D. jamesoni jamesoni*, *D. jamesoni kaimosae*, and the Kenyan locality of *D. polylepis*. However, for the South African *D. polylepis*, the antivenom provided protection only against the flaccid-paralysis postsynaptic effects of venom, as evidenced by the restoration of contractile responses to ACh and CCh. SAIMR polyvalent antivenom, however, failed to neutralise spastic-paralysis presynaptic/synaptic neurotoxicity, as shown by the increase in twitch height (fasciculation) beyond 100%. The neutralisation of spastic-paralysis presynaptic/synaptic and flaccid-paralysis postsynaptic *Dendroaspis* venom effects by antivenom is summarised in Table 2.

### 2.2. Molecular Phylogenetics and Evolution

**Figure 3:**
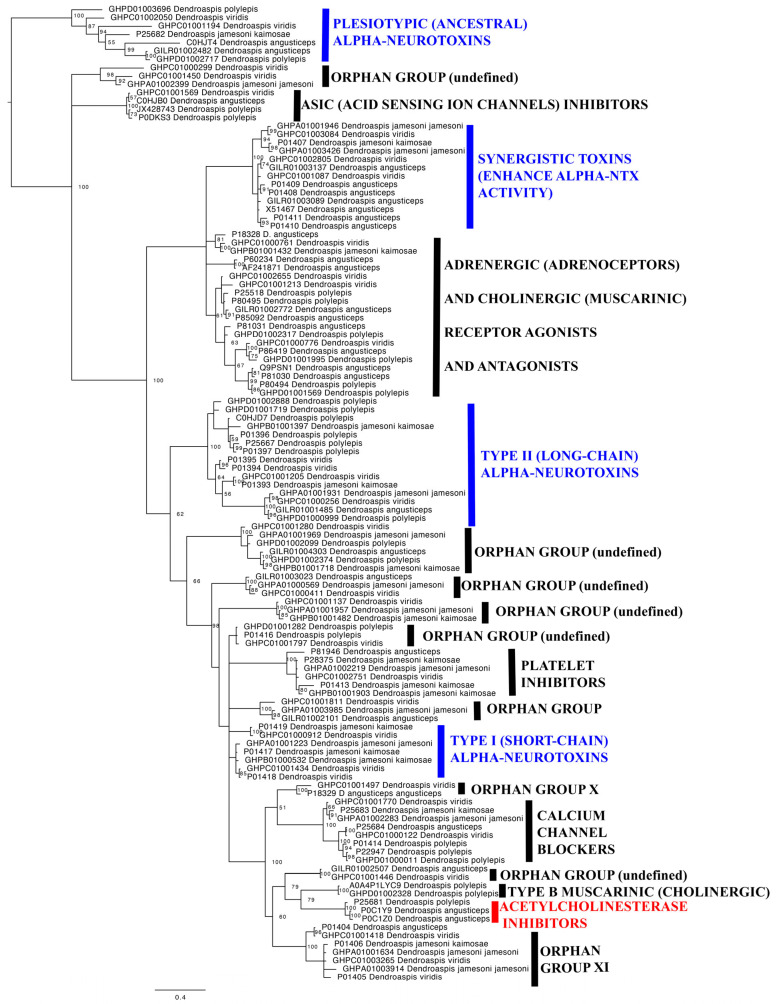
Molecular phylogenetics and evolution of 3FTx sequenced from *Dendroaspis* venoms. Orphan Group (undefined) refers to novel Orphan Groups revealed in this study. Other Orphan Group X and XI annotations are based on previous characterisations [27]. The tree was rooted using the *Dendroaspis* plesiotypic alpha-neurotoxins as the outgroups, as these represent the ancestral 3FTx type. Characterised flaccid-paralysis toxin types are in blue, and spastic-paralysis in red.

**Figure 4:**
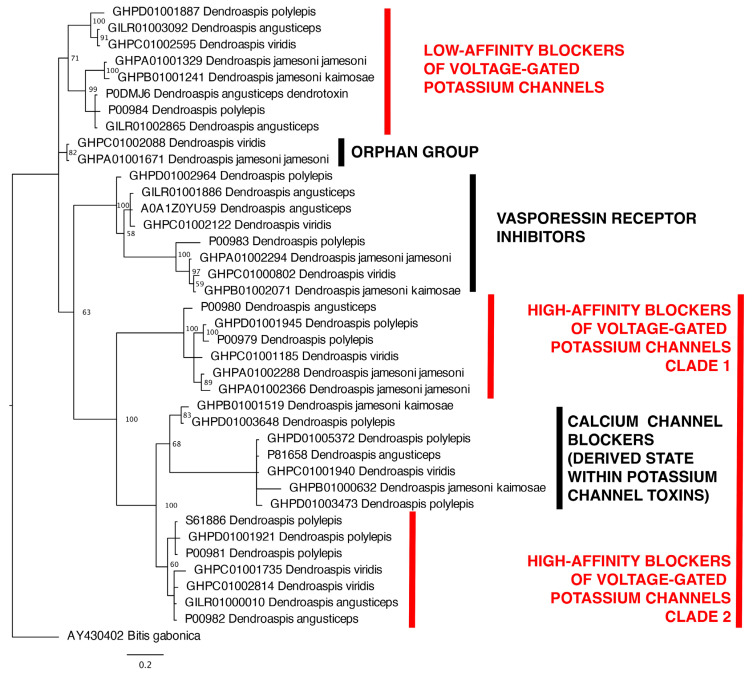
Molecular phylogenetics and evolution of kunitz-toxins sequenced from *Dendroaspis* venoms. Orphan Group is a toxin clade for which no bioactivity has been ascertained. Outgroup was AY430402 *Bitis gabonica* (removed from final processed image to avoid confusion). Characterised spastic-paralysis toxin types are shown in red.

To understand the molecular diversity of mamba venom peptides, and how this contributes to the observed differential spastic-paralysis presynaptic/synaptic and flaccid-paralysis postsynaptic effects, we reconstructed the molecular evolutionary history of the two dominant peptidic toxin types involved in neurotoxicity: 3FTx (three finger toxins) (Figure 3) and kunitz-toxins (Figure 4).

For the 3FTx, in addition to well-supported clades with known activities, we recovered nine Orphan Groups, which are defined as a phylogenetically distinct clade for which no known bioactivity is known. Of these Orphan Groups, two were previously defined clades [27] but the others were novel clades revealed for the first time in this study.

Within the kunitz-toxins, only one Orphan Group was recovered. A novel finding in this study, however, was revealing that the calcium channel blocking kunitz-toxins are derived from within the potassium channel blocking toxins, rending the latter toxin type non-monophyletic. The emergence of a novel functional trait from within a clade has been seen in other species, such as the Type III potassium channel toxins in sea anemone venoms emerging from within a clade of sodium channel toxins [28]. Some of these toxins shown to lack defined activity have been previously shown to be in high abundance in the venoms. Within both the 3FTx and kunitz toxin classes, clades of particular subtypes are shared across all the species (Figure 3 and Figure 4). In addition, individual isoforms are identically expressed across multiple species.

Consistent with the lack of flaccid-paralysis postsynaptic activity, the toxins shown previously to be the dominant types in *D. angusticeps* venom are phylogenetically distinct in our analyses from those with described flaccid-paralysis inducing α-neurotoxic activity [8] and, instead, spastic-paralysis inducing toxins are the dominant toxin types present. The 3FTx toxin type with the highest level of expression in the venom is uniprot accession code P0C1Z0, which was phylogenetically placed in our analyses within the acetylcholinesterase inhibitors (Figure 3). Muscarinic Toxin-2 (uniprot accession P18328) is present also as a major toxin and is located within the adrenergic/cholinergic clade of our analyses (Figure 3). However, the activity of this toxin type would not be captured in our assays as it is a smooth muscle acting toxin, while our assays test for actions upon skeletal muscle [18,22]. Of the kunitz-toxins found in *D. angusticeps* venom, the α-dendrotoxin (uniprot accession P00980) is the most abundant, and in our analyses was phylogenetically located within clade 1 of the high-affinity blocker of voltage-gated potassium channel blocking toxins (Figure 4). Dendrotoxin-K (uniprot accession P00981) is also present as one of the major kunitz-toxins and was shown in our molecular phylogenetics to be a clade 2 member of the high-affinity blocker of voltage-gated potassium channel blocking toxins (Figure 4). Another kunitz toxin present in significant amounts is uniprot accession code P25684, which is an L-type calcium channel blocker toxin in our phylogenetics (Figure 3). However, the venoms have been previously shown to include major toxins [8] that in our phylogenetics were shown to be located within Orphan Groups. These include uniprot accession code P01404, which was phylogenetically located Orphan Group XI (Figure 3). Another high abundance toxin was uniprot accession code P18329, located within Orphan Group X (Figure 3).

*D. j. jamesoni*, *D. j. kaimosae*, and *D. viridis* venoms have been shown to be quite similar to each other, with the dominant 3FTx toxin type being uniprot accession P01406, which is a member of the Orphan Group XI clade in our phylogenetic analyses. The venoms were also similar in that the dominant kunitz-toxin type present (uniprot accessions P00979 and P00980) were members of the clade 1 high-affinity blockers of voltage-gated potassium channels in our phylogenetic analyses (Figure 4). These similarities in phylogenetic composition of the venom peptides present parallel the similar neurotoxicity effects and antivenom patterns (Figure 1 and Figure 2).

*D. polylepis* is the only mamba venom not dominated by 3FTx, instead having kunitz-toxins in higher concentrations [8]. 3FTx may have spastic-paralysis presynaptic/synaptic or flaccid-paralysis postsynaptic neurotoxic sites of action, but only spastic-paralysis presynaptic/synaptic neurotoxicity has been documented for kunitz-toxins. As shown by our functional testing (Figure 2), while α-neurotoxic 3FTx are present in proportionally lower amounts, this is offset by their high level of potency. As such, the proteomics data are in agreement with the functional testing results in this study, where the dominant action is that of flaccid-paralysis postsynaptic blockage of the nicotinic acetylcholine receptor, but with potent spastic-paralysis presynaptic/synaptic activity leading to neurotransmitter release unmasked by the antivenoms (Figure 2). The dominant kunitz-toxins previously recorded were uniprot accession codes P00979 and P00981, which our phylogenetic analyses revealed to be clade 1 and clade 2 (respectively) high-affinity blockers of voltage-gated potassium channels (Figure 4).

We next examined the relative rate of evolution of the different clades of *Dendroaspis* toxins within the 3FTx and kunitz-toxin types. While, overall, significant pervasive signatures of natural selection (positive or negative) were not present in either 3FTx or kunitz-toxin sets, episodic selection was a consistent trait (Table 3). This was particularly the case for the plesiotypic and Type II (long-chain) α-neurotoxins, and the adrenergic + cholinergic neurotoxins within the 3FTx, but with little to no overlap between MEME and FUBAR results (Table 3). The only exceptions were the synergistic 3FTx clade and vasopressin clade within the kunitz-toxins, which showed fewer sites under episodic positive selection than any other dataset within their respective families (Table 3).

## 3. Discussion

Our data show that *Dendroaspis* venoms in general display potent flaccid-paralysis postsynaptic neurotoxicity as the dominant activity, producing flaccid-paralysis through antagonistic binding to the nicotinic acetylcholine receptor, thereby preventing the docking of the neurotransmitter acetylcholine (Figure 1 and Figure 2). The exception to this was *D. angusticeps*, which triggered spastic-paralytic effects (Figure 1). These functional results were congruent with the previous proteomics and transcriptomics results, which detailed the broad trends in regard to toxin types present [8,9,10,11]. However, antivenom testing revealed the picture to be more complex, with the non-*angusticeps* venoms containing spastic-paralysis presynaptic/synaptic spastic-paralysis inducing toxins in addition to the postsynaptic flaccid-paralysis inducing toxins. While these toxins are indeed present in other *Dendroaspis* species, their functional effects are often masked in in vitro preparations due to the rapid action and potency of α-neurotoxins. These α-neurotoxins bind to nicotinic acetylcholine receptors and rapidly abolish neuromuscular transmission, resulting in the loss of electrically evoked twitches in an organ bath assay. As a result, even though spastic-paralysis presynaptic/synaptic toxins, such as dendrotoxins, can enhance neurotransmitter release, their effects are not readily observable in vitro as they are masked by the activity of flaccid-paralysis postsynaptic neurotoxins. However, as the antivenoms were effective in neutralising the flaccid-paralysis postsynaptic neurotoxins, but not the spastic-paralysis presynaptic/synaptic neurotoxins, the antivenom administration resulted in spastic-paralytic presynaptic effects equal to or exceeding that induced by *D. angusticeps* venom. This is cause for clinical concern as antivenom administration may trade one type of paralysis (flaccid) for another (spastic). These results showing that non-*angusticeps Dendroaspis* venoms are able to produce a myriad of spastic-paralytic presynaptic and flaccid-paralytic postsynaptic effects (Figure 1 and Figure 2), and that the venoms contain diverse toxins within the major classes of 3FTx and kunitz-toxins (Figure 3 and Figure 4), are congruent with the clinical pictures of *Dendroaspis* envenomings being complex and neurologically multifactorial [5,6].

Our data demonstrate that the flaccid-paralysis postsynaptic activity driven by 3FTx is successfully neutralised by all three antivenoms used in this study. This is highlighted by the prevention of both twitch inhibition and decreased responses to exogenous agonists ACh and CCh. However, the flaccid-paralysis postsynaptic neurotoxicity caused by the venom from South African population of *D. polylepis* was not neutralised by the Inoserp Pan African antivenom, with twitches still being abolished over the 60-min experiment duration, although twitch inhibition was slightly delayed compared to venom alone. Rather than inefficacy of the antivenom, these data suggest that for this antivenom the South African population would require proportionally more antivenom than the Kenyan population.

The venoms from *Dendroaspis* species have been previously shown to increase responses to indirect stimulation in the chick biventer cervicis nerve muscle preparation as a result of spastic-paralysis presynaptic/synaptic neurotoxins [29], and this activity has indeed been shown in our study. However, due to the potency of α-neurotoxins present in *Dendroaspis* venoms, this augmentation in twitch response was only revealed after the neutralisation of flaccid-paralysis postsynaptic effects using various antivenoms. Our study also revealed varying results with regard to the protective effects of antivenom against spastic-paralysis presynaptic/synaptic or flaccid-paralysis postsynaptic activity. Generally, it was observed that the Premium Pan-African antivenom, which contains antibodies against *D. angusticeps*, *D. viridis*, *D. polylepis*, and *D. jamesoni* venoms, was able to neutralise the flaccid-paralysis postsynaptic effects of all aforementioned *Dendroaspis* venoms. In contrast, this antivenom was shown to be ineffective in protecting against the spastic-paralysis presynaptic/synaptic effects of venom, where present, resulting in twitches exceeding the height of the control. The Inoserp Pan African antivenom successfully protected against the flaccid-paralysis postsynaptic effects in all species except for the Kenyan locality of *D. polylepis*, where twitch augmentation was revealed. Although the flaccid-paralysis postsynaptic effects of the South African population were only partially protected by the Inoserp antivenom, it is unclear in this study whether the activity of spastic-paralysis presynaptic/synaptic neurotoxins was neutralised. Considering that the Inoserp Pan African antivenom contains immunogens to *D. polylepis* venom, it is likely that the antibody mixture is not raised against all *D. polylepis* from different localities and hence does not cover the wide dispersion of *D. polylepis* across sub-Saharan Africa.

The SAIMR antivenom provided successful protection against the effects of *D. polylepis* (Kenya), *D. j. jamesoni*, *D. j. kaimosae*, and *D. viridis* venoms. In contrast, the neutralisation of flaccid-paralysis postsynaptic effects also unmasked the spastic-paralysis presynaptic/synaptic activity of *D. polylepis* (South Africa) venom, as indicated by the twitch augmentation compared to control tissues. This reveals that, while the SAIMR antivenom has efficacy against the flaccid-paralysis postsynaptic neurotoxins, spastic-paralysis presynaptic/synaptic neurotoxins are not neutralised. Aligning with the results from our study, a previous case report from the Czech Republic reported that generalised fasciculations (which are caused by spastic-paralysis toxins) persisted within a patient envenomed by a captive *D. polylepis*, despite being administered two doses of SAIMR antivenom [30]. Other clinical studies and case reports have praised the effectiveness of the SAIMR antivenom in combination with early aggressive treatment [26,31,32]. Given the wide distribution of *D. polylepis*, this species is included in the immunising mixture of all three antivenoms included within this study. However, the differences in neutralisation between localities shown in our study may be a result of underlying geographic venom variation in the immunizing venoms. The SAIMR antivenom effectively covered the spastic-paralysis presynaptic/synaptic and the flaccid-paralysis postsynaptic activities of the Kenyan locality but not the spastic-paralysis presynaptic/synaptic effects of the South African sample in our study. As such, the *D. polylepis* specimen in the case report, where spastic-paralytic effects emerged subsequent to the neutralisation of the flaccid-paralytic effects [30], may have derived from a locality (such as South Africa) in which the spastic-paralysis venom effects are less effectively neutralised by the antivenom. In contrast, a *D. polylepis* case report that reported neutralisation of flaccid-paralytic effects, but without the emergence of secondary spastic-paralysis, was from a specimen of unstated locality but with this pattern consistent with our results for the Kenyan locality treated with the SAIMR polyvalent antivenom [33]. This disparity in effectiveness of the antivenom efficacy warrants further investigation into potential geographic variations in *D. polylepis* venom, which may inform antivenom strategies.

In contrast to other *Dendroaspis* species, *D. viridis* venom was effectively protected by all antivenoms despite not being in the immunising mixture of the SAIMR or Inosan antivenom, indicating a high conservation of toxins between *Dendroaspis* species. This corroborates previous antivenomics studies, in which *D. viridis* venoms were well neutralised by not only the antivenoms included in this study but also other commercially available African antivenoms [8]. Further investigations into the partially protected species’ venom effects are warranted, focusing on higher volumes or concentrations of antivenom. In addition, reversal studies have previously been conducted using the chick biventer assay and may be a relevant area of research with *Dendroaspis* species to provide an indicator for time dependant neutralisation of antivenoms to protect neurotoxic venom activity [34,35].

Differences in the twitch and AUC significance may be due to lower replicates (*n* = 3) and limiting the experiment observations to 60 min. Higher replications and longer experiment times may allow for more discernible spastic-paralysis presynaptic/synaptic activity [36,37], as increasing twitches caused by venom were trending upwards by the end of our 60-min experiment. Longer assay times may also allow for observation of twitch abolishment, as the available neurotransmitter would have been further depleted, leading to paralysis.

The peculiar pattern of neurotoxic activity displayed by mamba venoms raises questions about the evolutionary trajectory of neurotoxins in *Dendroaspis*. Our molecular evolution tests returned insightful results. The stark difference in number of sites experiencing pervasive vs. episodic positive selection (with a large bias towards the latter) indicates unequal rates of evolution not only across sites in toxin sequences but also between different toxins. Thus, it is possible that some 3FTx and kunitz-type toxins in *Dendroaspis* are experiencing rapid diversification in line with the general paradigm associated with snake venom proteins [27,38,39], whereas others have undergone fixation at an optimum for evolutionary advantage or are evolving under relaxed selection pressures in the first place.

Although venom evolution is independent of speciation and organismal phylogeny [40,41], the relatively recent diversification of mambas is in line with spikes of positive selection in a highly functional trait such as venom [3,42]. It is notable that all the clades within each toxin class are shared across all the species (Figure 3 and Figure 4). This is consistent with highly expressed individual isoforms identically expressed across multiple species [8]. This suggests that the *Dendroaspis*’ last common ancestor underwent early punctuated molecular evolution in the venom that paralleled the evolution of the unique morphotype that characterises mambas, with a slowing down of venom diversification subsequent to niche specialisation prior to speciation within the genus. This ‘two speed’ approach to venom evolution has been documented in other venomous lineages [42,43]. Consequently, the venom variations seen today are due to expression-level differences rather than the evolution of novel venom phenotypes within a species. This early divergence of mamba venom is ecologically consistent with *Dendroaspis* being one of very few arboreal elapid genera worldwide and also, for biogeographic reasons, with the genus subjected to recent rapidly receding or expanding barriers separating species and populations [3]. Conversely, the smaller number of sites under episodic positive selection in the synergistic 3FTx clade might be due to their complex interactions with other toxins, which would impose a further constraint on natural selection so as not to impair mutual enhancement of toxicity. A larger sample of mamba 3FTx and kunitz-type toxins for further phylogenetic and molecular evolution testing is unlikely to eventuate, as the available sequences are the result of intense transcriptome sequencing of all the species [8] and therefore may represent the diversity of toxins within the genus.

It is notable, however, that the species which contain both spastic-paralysis presynaptic/synaptic and flaccid-paralysis postsynaptic toxins (*D. polylepis*, *D. jamesoni*, and *D. viridis*) are not monophyletic relative to the species relationships within *Dendroaspis* of (*D. angusticeps* + *D. polylepis*) + (*D. jamesoni* + *D. viridis*). This suggests that *D. angusticeps*, with a venom containing only spastic-paralysis presynaptic/synaptic toxins (as shown by Figure 1 for bioactivity and also [8] for proteomics and transcriptomics), represents a derived state within the genus. As to why this species, which does not differ appreciably in ecology or morphology from the other two green coloured forest dwelling species *D. jamesoni* and *D. viridis*, it remains to be elucidated what selection pressures led to this unique venom phenotype.

## 4. Conclusions

This genus-wide evaluation of *Dendroaspis* venom neurotoxicity and antivenom efficacy highlights the complex interplay between species-specific venom effects, driven by the molecular evolutionary history of the toxins and therapeutic outcomes. The distinctive neurotoxic profile of *D. angusticeps*, as well as the synergistic spastic-paralysis presynaptic/synaptic and flaccid-paralysis postsynaptic activities of other *Dendroaspis* venoms represent a unique clinical challenge that is poorly addressed by current antivenom formulations. While all three tested antivenoms demonstrated efficacy against flaccid-paralysis postsynaptic neurotoxic effects, their limited capacity to neutralise the spastic-paralytic effects of presynaptic and acetylcholine-potentiating toxins represents a critical gap in current therapeutic coverage. As the study examined crude venoms, rather than pure toxins, it was not able to resolve whether the antivenom failure against the presynaptic/synaptic toxins that produces spastic-paralytic effects is in the 3FTX or kunitz-peptide toxin families, or both. Therefore, future work should examine purified toxins for their relative sensitivity to antivenoms. The intra-species variations in antivenom efficacy against *D. polylepis* venoms reveal additional issues, which may be paralleled by similar variations within the other three species This study has added to the growing body of knowledge surrounding *Dendroaspis*, toxin molecular phylogenetics, and evolution, and may help improve the clinical management of envenomation by these species.

## 5. Materials and Methods

### 5.1. Venoms and Antivenoms

Venom from four *Dendroaspis* species purchased from Latoxan, Valence, France, were used within this study; *D. angusticeps*, two localities of *D. polylepis* (Kenya and South Africa), and *D. viridis*, as well as both subspecies of *D. jamesoni*, *D. jamesoni jamesoni*, and *D. jamesoni kaimosae*. All venoms are pools of adult samples to minimise individual variations. Lyophilised venoms were reconstituted in 0.05% BSA to a working stock concentration of 2 mg/mL and stored at − 20 °C until use. Three antivenoms commercially available in Africa (Table 4) South African Institute for Medical Research (SAIMR)/South African Venom Producers (SAVP), polyvalent antivenom Sandringham, Johannesburg (Lot# L01146, expiry March 2002), Inoserp Pan African™ from Inosan Biopharma, S.A., Madrid, Spain (1IT07002, manufactured July 2021, expiry July 2024), and Snake Venom Antiserum (Pan Africa premium) from Premium Serums and Vaccines Pvt. Ltd., Junnar, Maharashtra, India (Panaf-033, manufactured May 2024, expiry April 2028). Antivenoms were made up to manufacturers’ instructions and 200 µL of each antivenom was used in the organ bath. Antivenoms have been shown to be stable over time, active for at least 60 years. As such, the use of expired antivenoms was not considered a concern in this study [44,45,46].

### 5.2. Drugs and Reagents

The following chemicals and drugs were purchased from Sigma-Aldrich, St Louis, MO, USA: acetylcholine (ACh), carbamylcholine (CCh), d-tubocurarine (dTC), and bovine serum albumin (BSA). Potassium chloride (KCl) was purchased from Merck, Darmstadt, Germany. All chemicals were dissolved in deionised water.

### 5.3. Chick Biventer Cervicis Nerve-Muscle Preparation

Animals were euthanased via CO_2_ inhalation. Methods used to test neurotoxic activity and antivenom efficacy reflect previously validated protocols [35,47,48]. In brief, two biventer muscles were dissected from the neck of each chick and mounted vertically on a wire holder under a 1 g resting tension in 5 mL organ baths. Tissues were maintained at 34 °C in a physiological salt solution (118.4 mM of NaCl, 4.7 mM of KCl, 1.2 mM of MgSO_4_, 1.2 mM of KH_2_PO_4_, 2.5 mM of CaCl_2_, 25 mM of NaHCO_3_, and 11.1 mM of glucose) and bubbled with carbogen (95% O_2_ and 5% CO_2_). Motor nerves were stimulated with a supramaximal voltage of 10–15 V (0.1 Hz; 0.2 ms) using an ADInstruments LE series electrical stimulator, Bella Vista New South Wales, Australia, Twitches were then recorded using a PowerLab system (ADInstruments Pty Ltd., Bella Vista, Australia) via a Grass FT03 force transducer. Tissues were allowed to equilibrate over a period of 20 min. To test for selective nerve stimulation, dTC (10µM) was added to the bath to ensure twitches were abolished. The dTC was then repeatedly washed from the bath using the physiological solution to restore twitches. In the absence of indirect nerve stimulation, contractile responses to exogenous ACh (1 mM, 30 s), CCh (20 µM, 60 s), and KCl (40 mM, 30 s) were obtained before the addition of venom and at the conclusion of the experiment. For antivenom neutralisation studies, antivenom was added to the bath 15 min before the addition of venom to ensure the antivenom alone had no inhibitory effect upon twitches. Experiments were conducted over 60 min.

This study was conducted according to the NHMRC Australian Code for the Care and Use of Animals for Scientific Purposes and approved by the Monash University Animal Ethics Committee. Animal ethics number: 26830, approved 30 April 2021.

### 5.4. Computational Molecular Evolution

Protein sequences of known 3FTx toxins and kunitz-type toxins from *Dendroaspis* were retrieved from UniProt and GenBank and aligned in AliView v. 1.1.4. Bayesian phylogenetic inference was subsequently performed in ExaBayes (https://github.com/aberer/exabayes, accessed on 2 February 2025) using four independent runs of twenty Metropolis-coupled Markov chains each. The 3FTx dataset was run for 8,000,000 generations per run and the kunitz dataset for 20,000,000 generations, until convergence. See Supplementary Folder S1 for full details and all files

Subsequently, all monophyletic clades with at least four sequences were selected for tests of molecular evolution aimed towards identifying the strength and direction (or lack thereof) of natural selection on these toxins (see Supplementary Folder S1 for full details and all files). The corresponding mRNA sequences for all such clades were sourced from GenBank and aligned in AliView as above, but corresponding phylogenies were constructed in IQtree v. 1.6.12 with 100 bootstrap replicates per dataset [49].

Three methods of determining natural selection signatures were employed in this study, all of which compute natural selection as the ratio between non-synonymous (dN) and synonymous (dS) substitutions in a sequence (dN/dS, commonly expressed with the parameter ω). Thus, ω > 1 represents positive (diversifying) selection, whereas ω < 1 indicates negative (purifying) selection, and ω = 1 stands for neutral evolution without significant directionality (i.e., genetic drift). First, each monopyletic clade of toxins was run in Fast Unconstrained Bayesian AppRoximation (FUBAR) [50]. This program computes ω values for each site but extends it across all branches of the phylogeny (i.e., pervasive selection with the same value for each site across sequences). The significance threshold for FUBAR was set to 95% posterior probability (PP) of selection.

By contrast, the Mixed Effects Model of Evolution (MEME) estimates episodic positive selection, whereby selection signatures are allowed to vary not only across sites but also across branches (i.e., the same site might show different selection signatures between sequences) [51]. For MEME, we adopted a significance threshold of α = 0.05. Lastly, a further test of episodic selection was carried out through Branch-Site Unrestricted Statistical Test for Episodic Diversification (BUSTED), which is known for its ability to accommodate small sample sizes, and compares the ω scores of a constrained model with ω > 1 not allowed against those of an unconstrained model where positive selection is permitted [52]. The BUSTED evidence ratio threshold to estimate positive selection (based on a likelihood ratio test of the constrained vs. unconstrained model) was set to 10.

### 5.5. Statistics

Twitch height was measured every 4 min after the addition of venom and expressed as a percentage of the pre-venom twitch height. Post-venom contractile responses to exogenous agonists were expressed as a percentage of the corresponding initial pre-venom contractile response. Data are presented as mean  ±  standard error of mean (SEM), where *n* is the number of tissue preparations. Significance was determined using a one-way ANOVA followed by Dunnett’s multiple comparisons. Comparisons of responses to exogenous agonists before and after venom treatment were performed using a Student’s paired *t*-test. All data and statistical analyses were performed using Prism 10.2.2 (GraphPad Software, San Diego, CA, USA, 2022). *p*  <  0.05 was considered statistically significant for all analyses.

## Figures and Tables

**Table 1:** Venom effects on twitch height. Potency is expressed as time (min) taken to reach 90% inhibition (t_90_) of the initial twitch response.

Species	t_90_ (min)
*D. angusticeps*	Not applicable
*D. jamesoni jamesoni*	24 ± 4
*D. jamesoni kaimosae*	17 ± 3
*D. polylepis* (Kenya)	24 ± 3
*D. polylepis* (South Africa)	15 ± 1
*D. viridis*	29 ± 6

**Table 2:** Summary of neutralisation of *Dendroaspis* spastic-paralysis presynaptic/synaptic and flaccid-paralysis postsynaptic effects by antivenom.

Species	Antivenom					
Neutralisation by:	SAIMR		Inoserp Pan African		Pan Africa Premium	
	Against Spastic- Paralysis	Against Flaccid- Paralysis	Against Spastic- Paralysis	Against Flaccid- Paralysis	Against Spastic- Paralysis	Against Flaccid- Paralysis
*D. angusticeps*	×	NA	Delayed	NA	×	NA
*D. jamesoni jamesoni*	✓	✓	✓	✓	×	✓
*D. jamesoni kaimosae*	✓	✓	✓	✓	×	✓
*D. polylepis* Kenya	✓	✓	×	✓	×	✓
*D. polylepis* South Africa	×	✓	NA	Partial	×	✓
*D. viridis*	-	✓	-	✓	-	✓

✓ = neutralised; × = not neutralised; NA = not applicable, - = not present.

**Table 3:** Number of sites showing significant signatures of natural selection (positive or negative) in BUSTED, FUBAR, and MEME for 3FTx and Kunitz-type toxins.

Three-Finger Toxins (3FTx)					
Clade	BUSTED	FUBAR (ω < 1)	FUBAR (ω > 1)	MEME	FUBAR (ω > 1) + MEME
Plesiotypic	14	0	2	9	1
Synergistic	6	1	0	2	0
Adrenergic + Cholinergic	10	0	1	7	0
Long chain	10	1	2	7	1
Orphan 1	15	0	0	2	0
Orphan 2	15	0	1	2	1
**Kunitz-type toxins**					
**Clade**	**BUSTED**	**FUBAR (ω < 1)**	**FUBAR (ω > 1)**	**MEME**	**FUBAR (ω > 1) + MEME**
Potassium channel blockers (high affinity)	9	0	2	4	0
Potassium channel blockers (low affinity)	1	1	1	2	1
Vasopressin	7	0	0	2	0

**Table 4:** Information regarding the immunising mixture of antivenoms from sub-Saharan Africa used in this study. Bold text indicates *Dendroaspis* species included in the immunising mixture of antivenoms.

Antivenom	Manufacturer	Immunising Mixture
South African polyvalent	South African Institute for Medical Research (SAIMR)	*Bitis arietans*
		*Bitis gabonica*
		***Dendroaspis angusticeps***
		***Dendroaspis jamesoni***
		***Dendroaspis polylepis***
		*Haemachetus haemachetus*
		*Naja annulifera*
		*Naja melanoleuca*
		*Naja mossambica*
		*Naja nivea*
Inoserp Pan African	Inosan Biopharma	*Bitis arietans*
		*Bitis gabonica*
		***Dendroaspis jamesoni***
		***Dendroaspis polylepis***
		*Echis leucogaster*
		*Echis ocellatus*
		*Echis pyramidum*
		*Naja haje*
		*Naja melanoleuca*
		*Naja nigricollis*
		*Naja pallida*
Pan African Premium	Premium Serums and Vaccines	*Bitis arietans*
		*Bitis gabonica*
		*Bitis nasicornis*
		*Bitis rhinoceros*
		*Echis carianatus*
		*Echis leucogaster*
		*Echis ocellatus*
		***Dendroaspis angusticeps***
		***Dendroaspis jamesoni***
		***Dendroaspis polylepis***
		***Dendroaspis viridis***
		*Naja haje*
		*Naja melanoleuca*
		*Naja nigricollis*

## Data Availability

The original contributions presented in this study are included in the article/Supplementary Materials. Further inquiries can be directed to the corresponding author(s).

## Supplementary Materials

The following supporting information can be downloaded from https://www.mdpi.com/article/10.3390/toxins17100481/s1, Supplementary Folder S1—Exabayes Files; Supplementary Folder S2—Molecular Analyses Files.
