# Supplementary figures and images for: Neurotoxic Sleight of Fang: Differential Antivenom Efficacy Against Mamba (Dendroaspis spp.) Venom Spastic-Paralysis Presynaptic/Synaptic vs. Flaccid-Paralysis Postsynaptic Effects
Source: Toxins (Basel). 2025 Sep 26;17(10):481. doi: 10.3390/toxins17100481 (PMC12568002; doi:10.3390/toxins17100481)

3FTx-set run-diagnostics

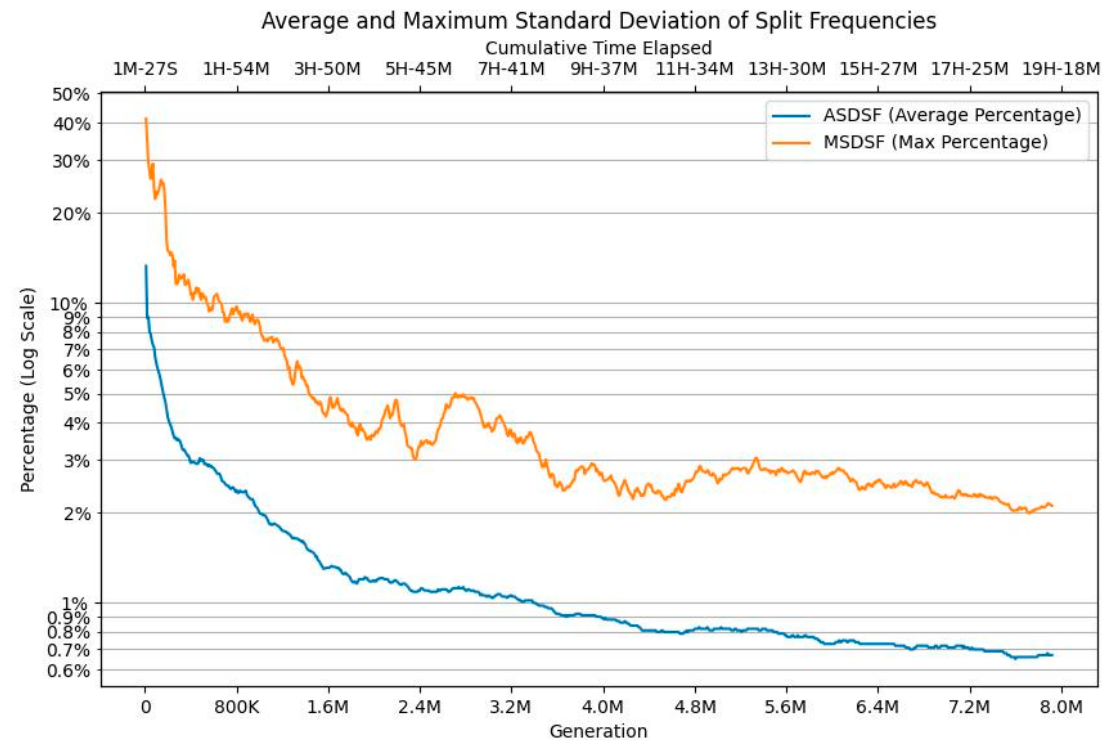

Kunitz-set run-diagnostics

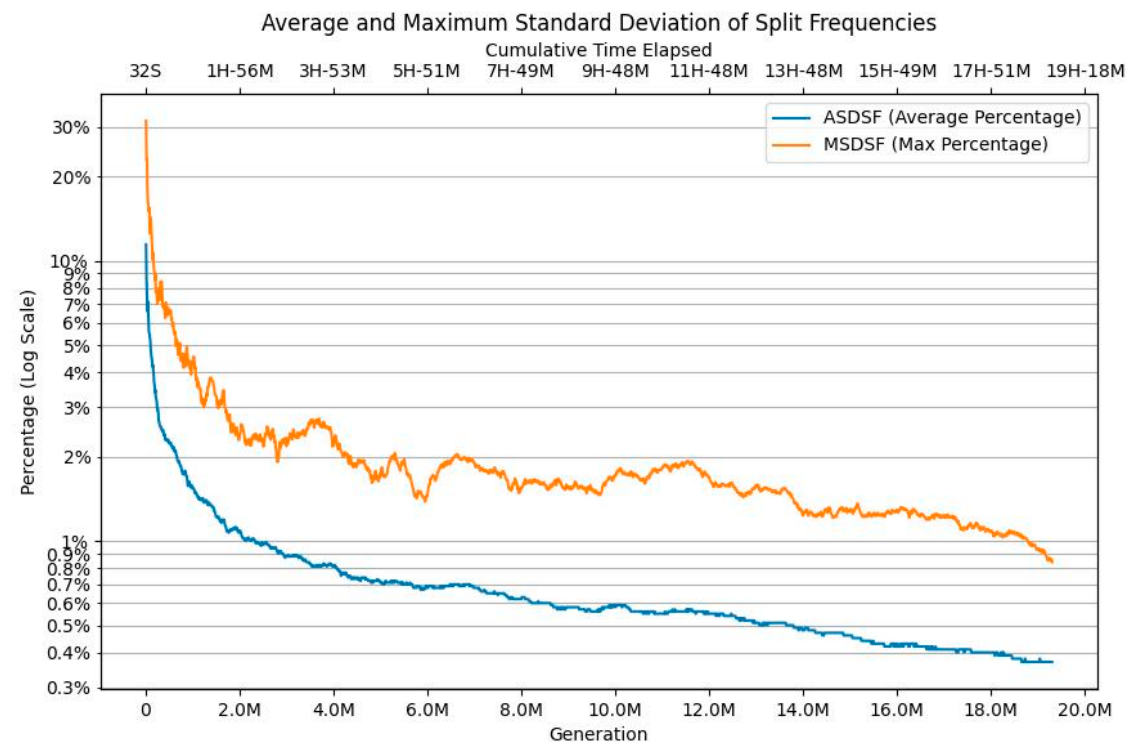

Supplement: Supplementary file 1 [file toxins-17-00481-s001.zip › Supplementary folder 1 - Exabayes/Run-set diagnostics.pdf]
